# Tripyridinium *cis*-tetra­chlorido­dioxido­molybdate(VI) chloride

**DOI:** 10.1107/S1600536810023950

**Published:** 2010-06-26

**Authors:** José A. Fernandes, Ana C. Gomes, Sónia Figueiredo, Sandra Gago, Paulo J. A. Ribeiro-Claro, Isabel S. Gonçalves, Filipe A. Almeida Paz

**Affiliations:** a Department of Chemistry, University of Aveiro, CICECO, 3810-193 Aveiro, Portugal; b Department of Chemistry, University of Algarve, Campus de Gambelas, 8005-139 Faro, Portugal

## Abstract

In the title compound, (C_5_H_6_N)_3_[MoCl_4_O_2_]Cl, the pyridinium cations are N—H⋯Cl hydrogen bonded to the anionic [MoCl_4_O_2_]^2−^ complexes and to the two crystallographically independent chloride anions (located on *C*2 axes). The Mo^6+^ centre adopts a highly distorted octa­hedral geometry, being surrounded by four chloride and two terminal oxide groups. The oxide ligands are mutually *cis*.

## Related literature

For a related structure, see: Luan *et al.* (2008 ▶). For previous studies by our group on dioxidomolybdenum complexes, see: Monteiro *et al.* (2010 ▶); Gago *et al.* (2009 ▶); Pereira *et al.* (2007 ▶); Cunha-Silva *et al.* (2007 ▶); Bruno *et al.* (2007 ▶). For graph-set notation for hydrogen-bonded aggregates, see: Grell *et al.* (1999 ▶). For a description of the Cambridge Structural Database, see: Allen (2002 ▶).
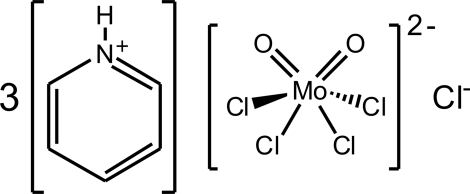


## Experimental

### 

#### Crystal data

(C_5_H_6_N)_3_[MoCl_4_O_2_]Cl
*M* *_r_* = 545.51
Trigonal, 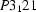

*a* = 11.3972 (2) Å
*c* = 29.4265 (9) Å
*V* = 3310.28 (13) Å^3^
*Z* = 6
Mo *K*α radiation
μ = 1.21 mm^−1^
*T* = 150 K
0.18 × 0.12 × 0.10 mm

#### Data collection

Bruker X8 Kappa CCD APEXII diffractometer
Absorption correction: multi-scan (*SADABS*; Sheldrick, 1998 ▶) *T*_min_ = 0.811, *T*_max_ = 0.888
33230 measured reflections
10395 independent reflections
8105 reflections with *I* > 2σ(*I*)
*R*_int_ = 0.047

#### Refinement

*R*[*F* ^2^ > 2σ(*F* ^2^)] = 0.038
*wR*(*F* ^2^) = 0.074
*S* = 1.05
10395 reflections
237 parameters
H-atom parameters constrained
Δρ_max_ = 0.46 e Å^−3^
Δρ_min_ = −0.68 e Å^−3^
Absolute structure: Flack (1983 ▶), 4490 Friedel pairs
Flack parameter: 0.03 (4)

### 

Data collection: *APEX2* (Bruker, 2006 ▶); cell refinement: *SAINT-Plus* (Bruker, 2005 ▶); data reduction: *SAINT-Plus*; program(s) used to solve structure: *SHELXTL* (Sheldrick, 2008 ▶); program(s) used to refine structure: *SHELXTL*; molecular graphics: *DIAMOND* (Brandenburg, 2006 ▶); software used to prepare material for publication: *SHELXTL*.

## Supplementary Material

Crystal structure: contains datablocks global, I. DOI: 10.1107/S1600536810023950/sj5018sup1.cif

Structure factors: contains datablocks I. DOI: 10.1107/S1600536810023950/sj5018Isup2.hkl

Additional supplementary materials:  crystallographic information; 3D view; checkCIF report

## Figures and Tables

**Table:** 

Mo1—O2	1.6988 (13)
Mo1—O1	1.7004 (13)
Mo1—Cl2	2.3750 (6)
Mo1—Cl1	2.3995 (6)
Mo1—Cl3	2.5746 (5)
Mo1—Cl4	2.5953 (5)

**Table:** 

O2—Mo1—O1	102.11 (7)
O2—Mo1—Cl2	94.45 (6)
O1—Mo1—Cl2	95.78 (6)
O2—Mo1—Cl1	94.06 (6)
O1—Mo1—Cl1	93.64 (6)
Cl2—Mo1—Cl1	165.71 (2)
O2—Mo1—Cl3	169.07 (5)
O1—Mo1—Cl3	88.75 (5)
Cl2—Mo1—Cl3	85.57 (2)
Cl1—Mo1—Cl3	83.91 (2)
O2—Mo1—Cl4	87.40 (5)
O1—Mo1—Cl4	170.36 (5)
Cl2—Mo1—Cl4	84.99 (2)
Cl1—Mo1—Cl4	83.94 (2)
Cl3—Mo1—Cl4	81.717 (16)

**Table 2:** Hydrogen-bond geometry (Å, °)

*D*—H⋯*A*	*D*—H	H⋯*A*	*D*⋯*A*	*D*—H⋯*A*
N1—H1*A*⋯Cl6^i^	0.88	2.16	3.0424 (19)	175
N2—H2*A*⋯Cl5^ii^	0.88	2.17	3.0304 (19)	166
N3—H3*A*⋯Cl4	0.88	2.45	3.243 (2)	150
N3—H3*A*⋯Cl3	0.88	2.69	3.277 (2)	126

Symmetry codes: (i) 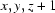; (ii) 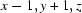.

**Fig. 1.**
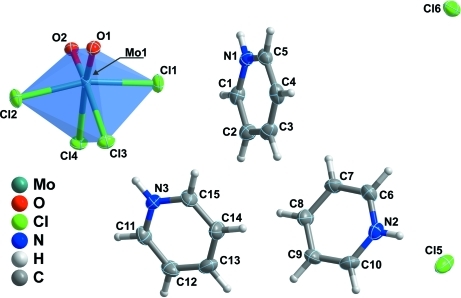
Schematic representation of the asymmetric unit. The [MoCl4O2]2- anion has a highly distorted {MoCl4O2} octahedral coordination environment, and the anions Cl5 and Cl6 are located on C2 axes. Thermal ellipsoids are drawn at the 50% probability level. For selected bond lengths (in Å) and angles (in degrees) see Table 1.

**Fig. 2.**
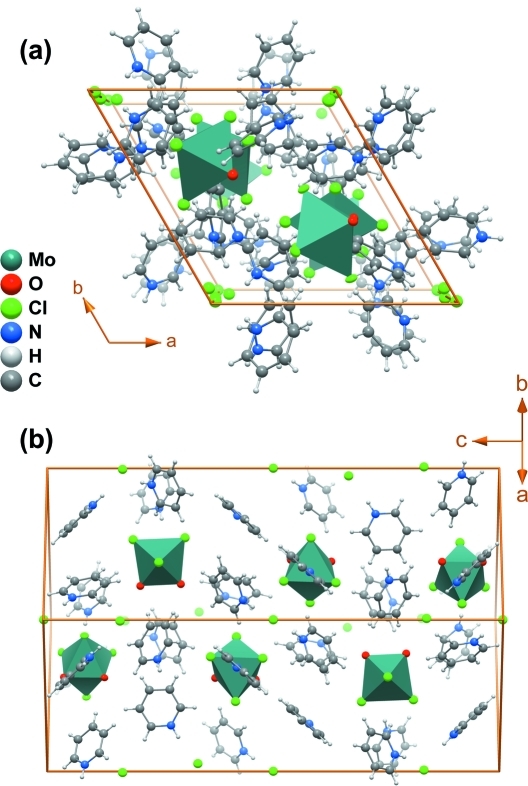
Crystal packing of the title compound viewed in perspective along the (a) [001] and (b) [110] directions of the unit cell.

## supplementary crystallographic information

### Comment

Our research group has been interested in the development of novel catalysts based on dioxomolybdenum complexes (Monteiro *et al.*, 2010; Gago *et al.*, 2009; Pereira *et al.*, 2007; Cunha-Silva *et al.*, 2007; Bruno *et al.*, 2007). During our recent efforts to coordinate pyridine to the molybdenum centre, we have isolated the title compound: [C_5_H_6_N]_3_[MoCl_4_O_2_]Cl. Remarkably, a search in the literature and in the Cambridge Structural Database (Allen, 2002) reveals the existence of only one other structure with the [MoCl_4_O_2_]^2-^ anion (Luan *et al.*, 2008).

The asymmetric unit of the title compound I is composed of three pyridinium (PyH) cations whose charge is balanced by the metallic dianion, [MoCl_4_O_2_]^2-^, and by two chloride anions located at special positions (Cl5 and Cl6 on C_2_ axes)  The Mo^6+^ centre is coordinated by four chloro and two oxo terminal ligands in a distorted octahedral geometry (Figure 1), {MoCl_4_O_2_}. The Mo—Cl distances range from 2.3750 (6) to 2.5953 (5) Å and the Mo═O distance is either 1.6988 (13) or 1.7004 (13) Å (Table 1). The *cis* and *trans* octahedral angles fall within a short range of the ideal values [81.717 (16)—103.11 (7)° and 165.71 (2)—170.36 (5)°, respectively - see Table 1].

The PyH cations are engaged in strong and relatively directional N^+^—H···Cl^-^ hydrogen bonds with the chloride anions (not shown; Table 2). We note that N3 is interacting with two spatially close chloro ligands (Cl3 and Cl4), forming a *R_1_^2^(4)* graph set motif (Grell *et al.*,1999) typical of bifurcated interactions. The crystal packing is, thus, mediated by the need to effectively fill the available space (Figure 2) since no significant supramolecular contacts are present in the crystal structure (*e.g.*, C—H···O or C—H···Cl contacts are all greater than 3.18 Å).

### Experimental

Chemicals were purchased from commercial sources and were used as received without purification.

To an aqueous solution (30 ml) of HCl (3.3 mol dm^-3^) containing 2.0 g (8.3 mmol) of Na_2_MoO_4_^.^2H_2_O, a solution of pyridine (1.34 ml, 16.6 mmol) in CH_2_Cl_2_ (60 ml) was slowly added dropwise. The biphasic mixture was vigorously stirred for 3 h at ambient temperature. The aqueous phase was separated and washed three times with CH_2_Cl_2_, and then allowed to evaporate yielding a solid. Crystals of the title compound were ultimately isolated by slow diffusion of diethyl ether into a concentrated solution in acetonitrile.

Crystals of the title compound were ultimately isolated by slow diffusion of diethyl ether into a concentrated solution in acetonitrile. Yield: 65%.

^1^H NMR (300.13 MHz, 298 K, CD_3_CN): δ = 8.75 (*d*, 2H), 8.56 (*t*, 1H), 8.03 (*t*, 2H) p.p.m..

Selected FT—IR (ATR, cm^-1^): 923 [*vs*, ν*_sym_*(Mo═O)], 884 [*vs*, ν*_asym_*(Mo═O)], 320 [*vs*, ν(Mo—Cl)].

### Refinement

Hydrogen atoms bound to carbon and nitrogen were located at their idealized positions and were included in the final structural model in riding-motion approximation with: C—H = 0.95 Å (aromatic) and N—H = 0.88 Å. The isotropic thermal displacement parameters for these atoms were fixed at 1.2 times *U*_eq_ of the respective parent atom.

A total of 4490 estimated Friedel pairs have not been merged and were used as independent data for the structure refinement. The Flack parameter (Flack, 1983) converged to 0.03 (4), ultimately assuring a correct absolute structure determination from the single-crystal data set.

### Crystal data

**Table:**

(C_5_H_6_N)_3_[MoCl_4_O_2_]Cl	*D*_x_ = 1.642 Mg m^−^^3^
*M**_r_* = 545.51	Mo *K*α radiation, λ = 0.71073 Å
Trigonal, *P*3_1_21	Cell parameters from 6207 reflections
Hall symbol: P 31 2"	θ = 5.5–33.1°
*a* = 11.3972 (2) Å	µ = 1.21 mm^−^^1^
*c* = 29.4265 (9) Å	*T* = 150 K
*V* = 3310.28 (13) Å^3^	Block, yellow
*Z* = 6	0.18 × 0.12 × 0.10 mm
*F*(000) = 1632	

### Data collection

**Table:**

Bruker X8 Kappa CCD APEXII diffractometer	10395 independent reflections
Radiation source: fine-focus sealed tube	8105 reflections with *I* > 2σ(*I*)
graphite	*R*_int_ = 0.047
ω & φ scans	θ_max_ = 36.3°, θ_min_ = 5.4°
Absorption correction: multi-scan (*SADABS*; Sheldrick, 1998)	*h* = −18→16
*T*_min_ = 0.811, *T*_max_ = 0.888	*k* = −17→8
33230 measured reflections	*l* = −48→49

### Refinement

**Table:**

Refinement on *F*^2^	Secondary atom site location: difference Fourier map
Least-squares matrix: full	Hydrogen site location: inferred from neighbouring sites
*R*[*F*^2^ > 2σ(*F*^2^)] = 0.038	H-atom parameters constrained
*wR*(*F*^2^) = 0.074	*w* = 1/[σ^2^(*F*_o_^2^) + (0.0224*P*)^2^ + 0.183*P*] where *P* = (*F*_o_^2^ + 2*F*_c_^2^)/3
*S* = 1.05	(Δ/σ)_max_ = 0.003
10395 reflections	Δρ_max_ = 0.46 e Å^−^^3^
237 parameters	Δρ_min_ = −0.68 e Å^−^^3^
0 restraints	Absolute structure: Flack (1983), 4490 Friedel pairs
Primary atom site location: structure-invariant direct methods	Flack parameter: 0.03 (4)

### Special details

**Table:**

Geometry. All e.s.d.'s (except the e.s.d. in the dihedral angle between two l.s. planes) are estimated using the full covariance matrix. The cell e.s.d.'s are taken into account individually in the estimation of e.s.d.'s in distances, angles and torsion angles; correlations between e.s.d.'s in cell parameters are only used when they are defined by crystal symmetry. An approximate (isotropic) treatment of cell e.s.d.'s is used for estimating e.s.d.'s involving l.s. planes.
Refinement. Refinement of *F*^2^ against ALL reflections. The weighted *R*-factor *wR* and goodness of fit *S* are based on *F*^2^, conventional *R*-factors *R* are based on *F*, with *F* set to zero for negative *F*^2^. The threshold expression of *F*^2^ > σ(*F*^2^) is used only for calculating *R*-factors(gt) *etc*. and is not relevant to the choice of reflections for refinement. *R*-factors based on *F*^2^ are statistically about twice as large as those based on *F*, and *R*- factors based on ALL data will be even larger.

### Fractional atomic coordinates and isotropic or equivalent isotropic displacement parameters (Å^2^)

**Table:**

	*x*	*y*	*z*	*U*_iso_*/*U*_eq_	
Mo1	0.663054 (16)	0.338004 (17)	0.915715 (4)	0.01643 (3)	
O1	0.77729 (15)	0.39544 (18)	0.87246 (4)	0.0238 (3)	
O2	0.76505 (15)	0.38526 (17)	0.96219 (4)	0.0233 (3)	
Cl1	0.64140 (7)	0.53687 (6)	0.916423 (17)	0.02869 (12)	
Cl2	0.62465 (7)	0.11310 (6)	0.914936 (17)	0.02893 (12)	
Cl3	0.47182 (5)	0.25297 (6)	0.856354 (14)	0.02601 (11)	
Cl4	0.45883 (5)	0.24295 (6)	0.971200 (14)	0.02623 (11)	
Cl5	1.00836 (8)	0.0000	0.8333	0.0421 (2)	
Cl6	0.94835 (8)	0.94835 (8)	0.0000	0.0352 (2)	
N1	0.6973 (2)	0.8428 (2)	0.94054 (6)	0.0266 (4)	
H1A	0.7726	0.8765	0.9564	0.032*	
N2	0.1156 (2)	0.8720 (2)	0.89938 (5)	0.0306 (4)	
H2A	0.0989	0.9161	0.8782	0.037*	
N3	0.2586 (2)	0.3111 (2)	0.91300 (7)	0.0333 (5)	
H3A	0.3299	0.3029	0.9186	0.040*	
C1	0.5793 (3)	0.7649 (3)	0.96154 (7)	0.0289 (5)	
H1	0.5782	0.7449	0.9929	0.035*	
C2	0.4601 (3)	0.7139 (3)	0.93800 (7)	0.0304 (5)	
H2	0.3759	0.6572	0.9526	0.037*	
C3	0.4649 (3)	0.7467 (3)	0.89223 (7)	0.0321 (5)	
H3	0.3832	0.7154	0.8756	0.039*	
C4	0.5876 (3)	0.8244 (3)	0.87119 (7)	0.0313 (6)	
H4	0.5916	0.8454	0.8398	0.038*	
C5	0.7049 (3)	0.8714 (3)	0.89623 (7)	0.0295 (5)	
H5	0.7907	0.9239	0.8821	0.035*	
C6	0.2380 (3)	0.9313 (3)	0.91843 (8)	0.0335 (6)	
H6	0.3064	1.0183	0.9085	0.040*	
C7	0.2645 (3)	0.8657 (3)	0.95249 (8)	0.0327 (6)	
H7	0.3501	0.9082	0.9673	0.039*	
C8	0.1656 (3)	0.7374 (3)	0.96502 (8)	0.0345 (6)	
H8	0.1833	0.6902	0.9882	0.041*	
C9	0.0411 (3)	0.6777 (3)	0.94404 (8)	0.0367 (6)	
H9	−0.0272	0.5887	0.9522	0.044*	
C10	0.0168 (3)	0.7481 (3)	0.91116 (8)	0.0345 (6)	
H10	−0.0693	0.7094	0.8969	0.041*	
C11	0.1483 (3)	0.2387 (3)	0.93686 (7)	0.0339 (6)	
H11	0.1464	0.1775	0.9592	0.041*	
C12	0.0362 (3)	0.2502 (3)	0.92996 (8)	0.0361 (6)	
H12	−0.0434	0.1989	0.9475	0.043*	
C13	0.0413 (3)	0.3393 (3)	0.89645 (9)	0.0395 (6)	
H13	−0.0356	0.3487	0.8908	0.047*	
C14	0.1574 (3)	0.4132 (3)	0.87173 (9)	0.0389 (7)	
H14	0.1618	0.4743	0.8490	0.047*	
C15	0.2673 (3)	0.3979 (3)	0.88024 (8)	0.0351 (6)	
H15	0.3485	0.4478	0.8633	0.042*	

### Atomic displacement parameters (Å^2^)

**Table:**

	*U*^11^	*U*^22^	*U*^33^	*U*^12^	*U*^13^	*U*^23^
Mo1	0.01527 (8)	0.01530 (8)	0.01882 (5)	0.00773 (7)	0.00036 (5)	0.00003 (5)
O1	0.0216 (7)	0.0270 (9)	0.0249 (5)	0.0137 (7)	0.0046 (5)	0.0054 (6)
O2	0.0215 (7)	0.0220 (9)	0.0248 (5)	0.0096 (7)	−0.0042 (5)	−0.0012 (6)
Cl1	0.0361 (3)	0.0210 (3)	0.0348 (2)	0.0187 (3)	−0.0008 (2)	−0.0004 (2)
Cl2	0.0381 (4)	0.0186 (3)	0.0334 (2)	0.0167 (3)	0.0018 (2)	−0.0006 (2)
Cl3	0.0213 (2)	0.0335 (3)	0.02208 (16)	0.0128 (2)	−0.00321 (16)	−0.00294 (19)
Cl4	0.0216 (2)	0.0311 (3)	0.02295 (17)	0.0109 (2)	0.00463 (16)	0.00273 (19)
Cl5	0.0355 (3)	0.0628 (7)	0.0371 (3)	0.0314 (3)	0.01181 (18)	0.0236 (4)
Cl6	0.0300 (3)	0.0300 (3)	0.0359 (3)	0.0077 (4)	−0.00797 (16)	0.00797 (16)
N1	0.0273 (10)	0.0259 (11)	0.0312 (7)	0.0169 (10)	−0.0071 (7)	−0.0074 (8)
N2	0.0337 (13)	0.0350 (13)	0.0280 (7)	0.0208 (10)	−0.0009 (8)	0.0038 (8)
N3	0.0245 (12)	0.0311 (13)	0.0480 (11)	0.0166 (10)	0.0007 (9)	0.0044 (9)
C1	0.0393 (14)	0.0348 (14)	0.0213 (7)	0.0250 (12)	0.0004 (8)	−0.0004 (8)
C2	0.0297 (12)	0.0334 (14)	0.0304 (9)	0.0174 (11)	0.0053 (8)	−0.0008 (9)
C3	0.0338 (13)	0.0349 (15)	0.0303 (9)	0.0192 (12)	−0.0104 (8)	−0.0087 (9)
C4	0.0473 (16)	0.0272 (13)	0.0220 (8)	0.0206 (12)	−0.0018 (9)	−0.0011 (8)
C5	0.0323 (13)	0.0228 (13)	0.0340 (9)	0.0141 (11)	0.0068 (9)	0.0018 (9)
C6	0.0337 (15)	0.0233 (13)	0.0389 (11)	0.0108 (12)	0.0003 (10)	0.0010 (10)
C7	0.0234 (13)	0.0319 (14)	0.0363 (10)	0.0091 (11)	−0.0074 (9)	−0.0007 (10)
C8	0.0315 (14)	0.0366 (16)	0.0336 (10)	0.0157 (13)	0.0005 (10)	0.0110 (10)
C9	0.0247 (13)	0.0299 (14)	0.0458 (12)	0.0064 (11)	0.0035 (10)	0.0090 (11)
C10	0.0224 (13)	0.0401 (16)	0.0370 (11)	0.0126 (12)	−0.0048 (10)	−0.0032 (10)
C11	0.0409 (16)	0.0288 (13)	0.0285 (9)	0.0148 (12)	0.0000 (9)	0.0074 (9)
C12	0.0300 (14)	0.0321 (15)	0.0416 (11)	0.0122 (12)	0.0131 (11)	0.0031 (10)
C13	0.0250 (14)	0.0436 (18)	0.0508 (13)	0.0178 (14)	−0.0058 (11)	−0.0029 (12)
C14	0.0353 (15)	0.0410 (18)	0.0414 (12)	0.0200 (14)	−0.0006 (11)	0.0158 (12)
C15	0.0325 (15)	0.0338 (16)	0.0395 (11)	0.0169 (13)	0.0130 (10)	0.0128 (10)

### Geometric parameters (Å, °)

**Table:**

Mo1—O2	1.6988 (13)	C4—C5	1.379 (4)
Mo1—O1	1.7004 (13)	C4—H4	0.9500
Mo1—Cl2	2.3750 (6)	C5—H5	0.9500
Mo1—Cl1	2.3995 (6)	C6—C7	1.372 (4)
Mo1—Cl3	2.5746 (5)	C6—H6	0.9500
Mo1—Cl4	2.5953 (5)	C7—C8	1.377 (4)
N1—C1	1.335 (3)	C7—H7	0.9500
N1—C5	1.336 (3)	C8—C9	1.375 (4)
N1—H1A	0.8800	C8—H8	0.9500
N2—C6	1.332 (4)	C9—C10	1.371 (4)
N2—C10	1.338 (4)	C9—H9	0.9500
N2—H2A	0.8800	C10—H10	0.9500
N3—C11	1.310 (3)	C11—C12	1.363 (4)
N3—C15	1.349 (3)	C11—H11	0.9500
N3—H3A	0.8800	C12—C13	1.395 (4)
C1—C2	1.369 (3)	C12—H12	0.9500
C1—H1	0.9500	C13—C14	1.369 (4)
C2—C3	1.392 (3)	C13—H13	0.9500
C2—H2	0.9500	C14—C15	1.370 (4)
C3—C4	1.373 (4)	C14—H14	0.9500
C3—H3	0.9500	C15—H15	0.9500
			
O2—Mo1—O1	102.11 (7)	C5—C4—H4	120.4
O2—Mo1—Cl2	94.45 (6)	N1—C5—C4	119.6 (2)
O1—Mo1—Cl2	95.78 (6)	N1—C5—H5	120.2
O2—Mo1—Cl1	94.06 (6)	C4—C5—H5	120.2
O1—Mo1—Cl1	93.64 (6)	N2—C6—C7	119.4 (3)
Cl2—Mo1—Cl1	165.71 (2)	N2—C6—H6	120.3
O2—Mo1—Cl3	169.07 (5)	C7—C6—H6	120.3
O1—Mo1—Cl3	88.75 (5)	C6—C7—C8	119.2 (3)
Cl2—Mo1—Cl3	85.57 (2)	C6—C7—H7	120.4
Cl1—Mo1—Cl3	83.91 (2)	C8—C7—H7	120.4
O2—Mo1—Cl4	87.40 (5)	C9—C8—C7	120.0 (2)
O1—Mo1—Cl4	170.36 (5)	C9—C8—H8	120.0
Cl2—Mo1—Cl4	84.99 (2)	C7—C8—H8	120.0
Cl1—Mo1—Cl4	83.94 (2)	C10—C9—C8	119.2 (3)
Cl3—Mo1—Cl4	81.717 (16)	C10—C9—H9	120.4
C1—N1—C5	122.4 (2)	C8—C9—H9	120.4
C1—N1—H1A	118.8	N2—C10—C9	119.4 (2)
C5—N1—H1A	118.8	N2—C10—H10	120.3
C6—N2—C10	122.8 (2)	C9—C10—H10	120.3
C6—N2—H2A	118.6	N3—C11—C12	120.6 (2)
C10—N2—H2A	118.6	N3—C11—H11	119.7
C11—N3—C15	122.7 (2)	C12—C11—H11	119.7
C11—N3—H3A	118.6	C11—C12—C13	118.4 (3)
C15—N3—H3A	118.6	C11—C12—H12	120.8
N1—C1—C2	120.24 (19)	C13—C12—H12	120.8
N1—C1—H1	119.9	C14—C13—C12	119.9 (3)
C2—C1—H1	119.9	C14—C13—H13	120.0
C1—C2—C3	118.6 (2)	C12—C13—H13	120.0
C1—C2—H2	120.7	C13—C14—C15	119.2 (3)
C3—C2—H2	120.7	C13—C14—H14	120.4
C4—C3—C2	120.0 (2)	C15—C14—H14	120.4
C4—C3—H3	120.0	N3—C15—C14	119.1 (3)
C2—C3—H3	120.0	N3—C15—H15	120.4
C3—C4—C5	119.14 (19)	C14—C15—H15	120.4
C3—C4—H4	120.4		
			
C5—N1—C1—C2	−1.7 (4)	C7—C8—C9—C10	−0.9 (4)
N1—C1—C2—C3	−1.0 (4)	C6—N2—C10—C9	−0.3 (4)
C1—C2—C3—C4	2.5 (4)	C8—C9—C10—N2	1.6 (4)
C2—C3—C4—C5	−1.5 (4)	C15—N3—C11—C12	−1.3 (4)
C1—N1—C5—C4	2.8 (4)	N3—C11—C12—C13	1.0 (4)
C3—C4—C5—N1	−1.1 (4)	C11—C12—C13—C14	−0.5 (4)
C10—N2—C6—C7	−1.9 (4)	C12—C13—C14—C15	0.2 (5)
N2—C6—C7—C8	2.6 (4)	C11—N3—C15—C14	1.0 (4)
C6—C7—C8—C9	−1.2 (4)	C13—C14—C15—N3	−0.5 (5)

### Hydrogen-bond geometry (Å, °)

**Table:**

*D*—H···*A*	*D*—H	H···*A*	*D*···*A*	*D*—H···*A*
N1—H1A···Cl6^i^	0.88	2.16	3.0424 (19)	175
N2—H2A···Cl5^ii^	0.88	2.17	3.0304 (19)	166
N3—H3A···Cl4	0.88	2.45	3.243 (2)	150
N3—H3A···Cl3	0.88	2.69	3.277 (2)	126

Symmetry codes: (i) *x*, *y*, *z*+1; (ii) *x*−1, *y*+1, *z*.

## References

[bb1] Allen, F. H. (2002). *Acta Cryst.* B**58**, 380–388. 10.1107/s0108768102003890 12037359

[bb2] Brandenburg, K. (2006). *DIAMOND* Crystal Impact GbR, Bonn, Germany.

[bb3] Bruker (2005). *SAINT-Plus* Bruker AXS Inc., Madison, Wisconsin, USA.

[bb4] Bruker (2006). *APEX2* Bruker AXS Inc., Madison, Wisconsin, USA.

[bb5] Bruno, S. M., Balula, S. S., Valente, A. A., Paz, F. A. A., Pillinger, M., Sousa, C., Klinowski, J., Freire, C., Ribeiro-Claro, P. & Gonçalves, I. S. (2007). *J. Mol. Catal. A*, **270**, 185–194.

[bb6] Cunha-Silva, L., Monteiro, B., Pillinger, M., Gonçalves, I. S., Rocha, J. & Almeida Paz, F. A. (2007). *Acta Cryst.* E**63**, m376–m378.

[bb7] Flack, H. D. (1983). *Acta Cryst.* A**39**, 876–881.

[bb8] Gago, S., Neves, P., Monteiro, B., Pêssego, M., Lopes, A. D., Valente, A. A., Paz, F. A. A., Pillinger, M., Moreira, J., Silva, C. M. & Gonçalves, I. S. (2009). *Eur. J. Inorg. Chem.* pp. 4528–4537.

[bb9] Grell, J., Bernstein, J. & Tinhofer, G. (1999). *Acta Cryst.* B**55**, 1030–1043. 10.1107/s0108768199007120 10927445

[bb10] Luan, Y., Wang, G., Luck, R. L., Wang, Y., Xiao, H. & Ding, H. (2008). *Chem. Lett.* **37**, 1144–1145.

[bb11] Monteiro, B., Cunha-Silva, L., Gago, S., Klinowski, J., Paz, F. A. A., Rocha, J., Gonçalves, I. S. & Pillinger, M. (2010). *Polyhedron*, **29**, 719–730.

[bb12] Pereira, C. C. L., Balula, S. S., Paz, F. A. A., Valente, A. A., Pillinger, M., Klinowski, J. & Gonçalves, I. S. (2007). *Inorg. Chem.* **46**, 8508–8510. 10.1021/ic701746r 17883270

[bb13] Sheldrick, G. M. (1998). *SADABS* Bruker AXS Inc., Madison, Wisconsin, USA.

[bb14] Sheldrick, G. M. (2008). *Acta Cryst.* A**64**, 112–122. 10.1107/S0108767307043930 18156677

